# Role of Opioid Receptors Signaling in Remote Electrostimulation - Induced Protection against Ischemia/Reperfusion Injury in Rat Hearts

**DOI:** 10.1371/journal.pone.0138108

**Published:** 2015-10-02

**Authors:** Hsin-Ju Tsai, Shiang-Suo Huang, Meng-Ting Tsou, Hsiao-Ting Wang, Jen-Hwey Chiu

**Affiliations:** 1 Institute of Traditional Medicine, School of Medicine, National Yang-Ming University, Taipei, Taiwan, ROC; 2 Department of Pharmacology, School of Medicine, Chung Shan Medical University, Taichung, Taiwan, ROC; 3 Department of Family Medicine, Mackay Memorial Hospital, Taipei, Taiwan, ROC; 4 Division of General Surgery, Department of Surgery, Taipei Veterans General Hospital, Taipei, Taiwan, ROC; 5 Division of General Surgery, Department of Surgery, Cheng-Hsin General Hospital, Taipei, Taiwan, ROC; Emory University, UNITED STATES

## Abstract

**Aims:**

Our previous studies demonstrated that remote electro-stimulation (RES) increased myocardial GSK3 phosphorylation and attenuated ischemia/ reperfusion (I/R) injury in rat hearts. However, the role of various opioid receptors (OR) subtypes in preconditioned RES-induced myocardial protection remains unknown. We investigated the role of OR subtype signaling in RES-induced cardioprotection against I/R injury of the rat heart.

**Methods & Results:**

Male Spraque-Dawley rats were used. RES was performed on median nerves area with/without pretreatment with various receptors antagonists such as opioid receptor (OR) subtype receptors (KOR, DOR, and MOR). The expressions of Akt, GSK3, and PKCε expression were analyzed by Western blotting. When RES was preconditioned before the I/R model, the rat's hemodynamic index, infarction size, mortality and serum CK-MB were evaluated. Our results showed that Akt, GSK3 and PKCε expression levels were significantly increased in the RES group compared to the sham group, which were blocked by pretreatment with specific antagonists targeting KOR and DOR, but not MOR subtype. Using the I/R model, the duration of arrhythmia and infarct size were both significantly attenuated in RES group. The mortality rates of the sham RES group, the RES group, RES group + KOR antagonist, RES group + DOR/MOR antagonists (KOR left), RES group + DOR antagonist, and RES group + KOR/MOR antagonists (DOR left) were 50%, 20%, 67%, 13%, 50% and 55%, respectively.

**Conclusion:**

The mechanism of RES-induced myocardial protection against I/R injury seems to involve multiple target pathways such as Akt, KOR and/or DOR signaling.

## Introduction

Heart disease is the number one killer worldwide and caused the death of 7.3 million people in 2010 [1]. Ischemic heart disease (IHD), characterized by narrowed blood vessels and blockage of blood flow to the heart muscle, which finally causes a heart attack, is the most common form of heart disease. The major risk factors are high-fat diet, smoking, diabetes, high blood pressure and the genetic makeup of the individual [2, 3]. Although many therapies have been shown to bring about a significant reduction in mortality among myocardial infarction patients [4, 5], such beneficial effects are still of limited efficacy. As a result, new therapies are currently being investigated [6].

GSK-3, a Ser/Thr kinase, is an inactivator of the enzyme glycogen synthase and acts as a multifunctional downstream switch that regulates many transduction signalings [7, 8]. Dysregulated GSK-3 has been implicated in several diseases including type II diabetes, Alzheimer's disease, bipolar disorder, and cancer [9–12]. Recent studies demonstrated that catalytically-active GSK3 was implicated in anti-hypertrophic signaling [13]and that an inhibition of GSK3 resulted in changes in the activities of various transcription and translation factors found in the heart; furthermore, these change promoted hypertrophic responses [14]. In addition, it has been shown that selective inhibition of GSK has a similar effect to ischemic preconditioning (IPC) using isolated rat hearts; specifically, IPC was found to reduce GSK3-β activity by phosphorylating GSK3-β at the protein's N-terminal serine residue Ser9 [15]. However, cross talk between GSK3 and opioid-induced cardioprotection has not as yet been elucidated.

Opioids play an important role in protecting against ischemia/reperfusion (I/R) injury in many organs including the kidneys [16], central nerve system [17], and heart [18, 19]. Schultz et al., showed that naloxone, a non-selective opioid receptor antagonist, is able to block the cardioprotection afforded by brief periods of ischemia [20]. Accumulating evidence suggests that protein kinase Cε (PKCε) signaling is involved in this opioid receptor-dependent cardioprotection [21, 22]. Nevertheless, the linkage between GSK3, opioid receptors and remote electro-stimulation (RES) has not as yet been explored in any detail [23].

Recently, remote conditioning by ischemia or pharmacological agent was postulated to protect the heart against I/R injury [24, 25]. Furthermore, remote electro-stimulation (RES) on median nerve has been demonstrated to modulate the functions of the corresponding organ, for example the heart, in a variety of ways. Experimental studies have shown that RES is able to induce inhibition of cardiovascular sympathetic neurons that have been activated through visceral reflex stimulation; this activation is believed to occur via neurons in a number of regions of the brain, namely the arcuate nucleus of the hypothalamus, the vlPAG in the midbrain and the NRP in the medulla. These regions then, in turn, inhibit the activity of premotor sympathetic neurons in the rVLM [26, 27]. However, how RES affects the heart via GSK3- and opioid signaling remains unclear.

Previously, we demonstrated that RES protects rat heart against I/R injury.[28, 29] Furthermore, by proteomics analysis we found that RES induced phosphorylation of the GSK-3 protein [28]. It is generally accepted that AKT activation is an important aspect of pro-survival signaling related to myocardial protection. Nonetheless, there is little information available concerning how RES induces myocardial GSK-3 phosphorylation via the AKT/GSK-3 signaling pathway. Therefore, the aim of study was to investigate the mechanisms related to how RES affects myocardial ATK/GSK-3 signaling and then to explore opioid-receptor mediated myocardial protection.

## Materials and Methods

### Animals

Male Sprague-Dawley rats weighting 250–300 gm were obtained from the animal center of National Science Council, Taiwan, ROC, and were fed a standard diet and water *ad libitum*. All animals were treated under the regulations of the “Guide for the care and use of laboratory animals” (DHHS publication No. [NIH], revised 1996) and the“Improving bioscience research reporting for animal research”.[30]

The study was approved by the experimental animal committee of National Yang- Ming University (# 1001205). Zoletil (VIRBAC Laboratories, Carros, France, 50 mg/kg, IP) was used for anesthesia and the depth of anesthesia was maintained at a steady level that did not affect the rats' heart rate or arterial blood pressure, but also precluded any pain response induced by skin traction. The animals were used to carry out the experiments described below and eventually all animals were sacrificed using an overdose of Zoletil 50.

### Reagents

A number of adrenergic and cholinergic receptor antagonists or inhibitors were used in this study, namely the α-adrenergic receptor antagonist phentolamine (1.5 mg/kg), the β-adrenergic receptor antagonist propranolol (2 mg/kg) and the cholinergic (muscarinic) receptor (M2) antagonist atropine (0.016–0.032 mg/kg) together with a variety of antagonists targeting a range of opioid receptor subtypes, namely the non-specific opioid receptor antagonist naloxone (1.5mg/kg), the delta opioid receptor (DOR) antagonist naltrindole hydrochloride (NTI, 3.8 μmole/kg), the kappa opioid receptor (KOR) antagonist nor-binaltorphimine dihydrochloride (7.8 μmole/kg) and the mu opioid receptor (MOR) antagonist CTAP (0.18 μmole/kg). All the above chemicals were purchased commercially.

### Parameters used during electro-stimulation in the area of the median nerve

To study the sensory input involved in remote electro-stimulation (RES)-induced myocardial AKT/GSK-3 activation, RES was conducted bilaterally in the area of the median nerves. The parameters used for RES were set at a frequency of 2/15 Hz, alternatively, an intensity of 1–2 mA, a duration of 30 min and a pulse width of 400 μsec.[25]

### Study groups

**Fig 1:**
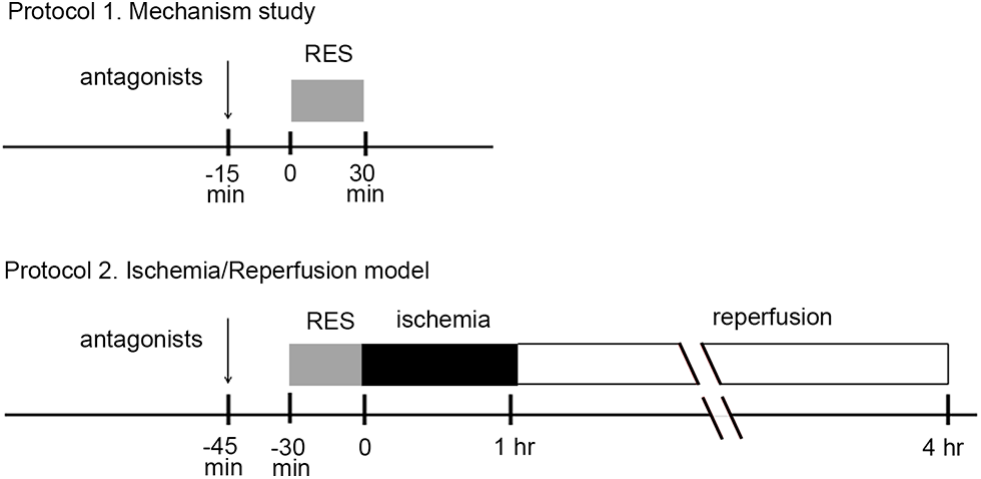
Study design and protocols. Two protocols were designed in this study, namely, 1) mechanistic study and 2) I/R model. In mechanistic protocol, various receptors antagonists were pretreated 15 min before remote electro-stimulation (RES). In I/R model, various receptors antagonists were pretreated 15 min before RES preconditioning, followed by 1h-ischemia and 3 h-reperfusion.

Two protocols were designed in this study, namely, 1) mechanistic study and 2) I/R model. In mechanistic protocol, various receptors antagonists were pretreated 15 min before RES preconditioning. In the I/R model, animals were randomly allocated into 6 groups. They were 1) sham RES group; 2) RES preconditioning group; 3) RES preconditioning pretreated with KOR blocker; 4) RES preconditioning pretreated with DOR blocker; 5) RES preconditioning pretreated with KOR/MOR antagonists, which left DOR active; 6) RES preconditioning pretreated with DOR/MOR antagonists, which left KOR active. In this protocol, various receptors antagonists were pretreated 15 min before RES preconditioning, followed by ischemia for 1 h and reperfusion for 3 h (Fig 1). The combination of receptor antagonists and their corresponding abbreviations were shown in Table 1.

**Table 1:** Drug and treatment information in this study.

Treatment	Abbreviations	Drug name
α-adrenoceptor antagonist		Phentolamine
β-adrenoceptor antagonist		Propranolol
Muscarinic receptors antagonist		Atropine
Delta opioid receptor antagonist	DOR blocker	Naltrindole hydrochloride
Kappa opioid receptor antagonist	KOR blocker	nor-Binaltorphimine dihydrochloride
Delta opioid receptor left	DOR left	nor-Binaltorphimine dihydrochloride +CTAP
Kappa opioid receptor left	KOR left	Naltrindole hydrochloride+CTAP
Mu opioid receptor left	MOR left	Naltrindole hydrochloride+nor-Binaltorphimine dihydrochloride

CTAP

### Sample preparation and protein concentration

After bilateral RES for 30 min in the area of the median nerve with or without pretreatment with the receptor antagonists or inhibitors at different time intervals (0 min, 30 min, 60 min), the animals were sacrificed immediately and their hearts were prepared for further analysis.

### Western blot analysis for myocardial proteins expression

Individual issue homogenates were prepared from the rat hearts in the presence of protease inhibitors on ice and the protein concentrations of these samples were obtained by Bradford's method [31]. Next, 30 μg of each homogenate was separated by 10% SDS-polyacrylamide gel electrophoresis and this was followed by transfer to a PVDF membrane. The membrane was then blocked with skimmed milk, which was followed by incubation with various different primary antibodies. The primary antibodies used were polyclonal rabbit anti-α-tubulin (1:1000, # 2144, Cell Signaling), rabbit anti-β-actin (1: 5000, Cell signaling), rabbit anti-p-GSK3α/β (Ser 21/9) (1:1000, Cell Signaling), rabbit anti-T-GSK3α/β (1:1000, AF-2157, R&D systems), rabbit anti-p-AKT (Ser473) (1:1000, #9217, Cell Signaling), rabbit anti-T-AKT (1:1000, #9272, Cell Signaling) and goat anti-p-PKCε (1:200, #SC12615, SANTA CRUZ), all of which were purchased commercially. After washing and incubation with 2,000-fold diluted biotin-conjugated secondary antibody (anti-goat, and anti-rabbit IgG: HRPO–Horseradish peroxidase, ME5345, Transduction Lab. Co., Lexington, KY, USA), the various proteins were detected using an enhanced chemiluminescence detection kit (ECL, Amersham Pharmacia Biotech., Inc.,NJ, USA) and analyzed by autoradiography. Monoclonal antibody against α-tubulin or β-actin was used as the internal control. The optical density (O.D.) values of the various bands were analyzed using a computer that was equipped with image analysis software (PhotoCapt, Vilber Lourmat, Marne LuVallee Cedex, France).

### Extraction of RNA, RT-PCR and the analysis of the RT-PCR products

Total RNA was isolated from the hearts by a modification of the single-step guanidinium thiocyanate method (TRI REAGENT, T-9424, Sigma Chem. Co., St. Louis, MO, USA) [32]. Reverse transcription was performed using a reverse transcription kit. Specifically, 1 μg of rat tissue RNA was used as a template and reverse transcription used to generate cDNA. Various genes, including glyceraldehyde-3-phosphate dehydrogenase (G3PDH) as the internal control, were then amplified by polymerase chain reaction (PCR). The primers used in this study. (Blossom Biotechnologies Inc., Eurogentec-AIT, Singapore) were as follows:

μ-opioid receptor (sense 5’-GAACAGCAAAACTCCACTCG-3’, anti-sense 5’-AGTTAGGGCAATGGAGCAGT-3’); κ-opioid receptor (sense 5’-GCATTTGGCTACTGGCATCA-3’, anti-sense 5’-GGAAACTGCAAGGAGCATTCA-3’) δ-opioid receptor (sense 5’-CAACGTGCTCGTCATGTTTGGA-3’, anti-sense 5’-CATCAGGTACTTGGCGCTCT-3’); GAPDH (sense 5’-AACTCCCTCAAGATTGTCAGCAA-3, anti-sense 5’-CAGTCTTCTGAGTGGCAGTGATG-3’)

The possible contamination of any PCR component was excluded by performing a PCR reaction using the same components, but in the absence of the RT product for each set of experiments (negative control). Finally, 20 μl of RT-PCR product was separated by 2% agarose gel electrophoresis in the presence of 0.2 μg/ml ethidium bromide, which was photographed using an ultraviolet transilluminator system.

### Immunofluorescent staining to detect the localization of the kappa opioid receptor

In order to localize kappa opioid receptor expression in the RES-treated hearts, paraffin-embedded tissue samples were de-paraffined with xylene and a serial concentrations of ethanol, cryosectioned (10 μm), fixed in 4% formaldehyde for 5 min and then this was followed by blocking with 2% bovine serum albumin (BSA) for another 1 h. Next the tissue sections were sequentially incubated with the following antibodies.1) Rabbit anti-κ opioid receptor (1:200, #9217, Cell Signaling) at room temperature for 1h followed by the secondary antibody Goat anti-rabbit Ig-FITC (1: 200, 711-095-152, Jackson ImmunoResearch, USA) for 1h; 2) Rabbit anti-rat VEGFR2 (KDR, 1:100X, #2479, Cell Signaling Technology Inc., MA) at room temperature for 1h followed by the secondary antibody (Donkey anti-rabbit IgG (H+L)-rodamine, 1: 50X, 711-026-152, Jackson ImmunoResearch Lab. Inc., PA) for 1 h. Finally, the tissue sections were stained with DAPI (4’,6-diamidino-2-phenylindole, 1:1000X, D-9542, Sigma-Aldrich Co., MO) for 1 min, the tissue slides mounted using 50% glycerol and observed under a fluorescence microscope (Zeiss, Germany).

### Animal models and the parameters used for ischemia/reperfusion injury of the heart

Ischemia/reperfusion (I/R) injury of the heart was performed as described previously but with some modification [33]. In brief, male SD rats were anesthetized with urethane (400 mg/kg i.p.) and placed on an operating table. After tracheotomy, in order to maintain normal pO2 and pH parameters, the animals were ventilated with room air by a small rodent respirator (model 131, NEMI, USA) with a stroke volume of 12 ml/kg body weight and at a rate of 60 strokes/min. The jugular vein was cannulated for drug administration and the carotid artery was cannulated for continuous monitoring of the animal's heart rate (HR) and arterial blood pressure (BP). After left thoracotomy, the 4th and 5th ribs were sectioned. The heart was then quickly externalized, inverted and a 6/0 silk ligature was placed around the left main coronary artery. The heart was repositioned in the chest and the animal was allowed to recover for 15 min. Animals in which the procedure produced arrhythmia or a sustained decrease in BP of less than 70 mmHg were excluded from this study. Following ischemia by ligating the left coronary artery (LAD) for 1 hr, reperfusion was achieved by releasing the tension applied to the ligature for a further 180 min.

### Infarction size

After 180 min of reperfusion, the coronary artery was re-occluded and Evan's blue (1%) was injected for 5 min into aortic artery to mark the area at risk. The non-ischemic area then appeared blue, while the area at risk remained unstained. Next the area at risk was excised, weighed and the occluded zone was expressed as the percentage of the total ventricular weight. Thereafter, the excised ventricular tissue was sliced into 1-mm sections and incubated with tetrazolium dye (2,3,5-triphenyltetrazolium chloride 1% (Sigma, USA) in normal saline) at 37°C for 15 min in darkness. Each section was then placed in a solution of 4% formaldehyde overnight and the infarcted tissue zone (white) measured. The weight of the infarcted tissue was expressed both as a percentage of the total ventricle and as a percentage of the area at risk [34].

### Hemodynamic index

Before and during the ischemia and reperfusion periods, HR, BP and ECG changes were recorded on computer using WAVE FORM data analysis software (MacLab data acquisition system, AD Instruments, Castle Hill, NSW, Australia). Ventricular ectopic activity was evaluated according to the diagnostic criteria advocated by the Lambeth Convention [20]. The number of ventricular premature beats (VPB) and the incidence and duration of ventricular tachyarrhythmias, including ventricular tachycardia (VT) and fibrillation (VF), among the surviving animals were determined

### CK-MB level in serum

At end of reperfusion, 2 ml arterial blood was collected and centrifuged at 1,500 × g. The serum was then collected and stored at −70°C prior to the measurement of the serum levels of CK-MB by enzyme-linked immunosorbent assay (ELISA).

### Statistics

Data were presented as mean ± S.E.M and were analyzed using GraphPad Prism V4.03 for Windows (GraphPad Software, San Diego California USA). Unpaired Student's t tests or Mann-Whitney *U* tests were used to analyze the differences between two groups; while concentration dependent and time effects were analyzed by repeatedly measured one-way analysis of variance (ANOVA) followed by Dunnet’s test. Mortality rate was analyzed using Fisher’s exact test. A *p* value less than 0.05 is considered statistically significant.

## Results

### The role of α-adrenergic receptor, β-adrenergic receptor and muscarinic receptor signaling in RES-induced Akt phosphorylation

**Fig 2:**
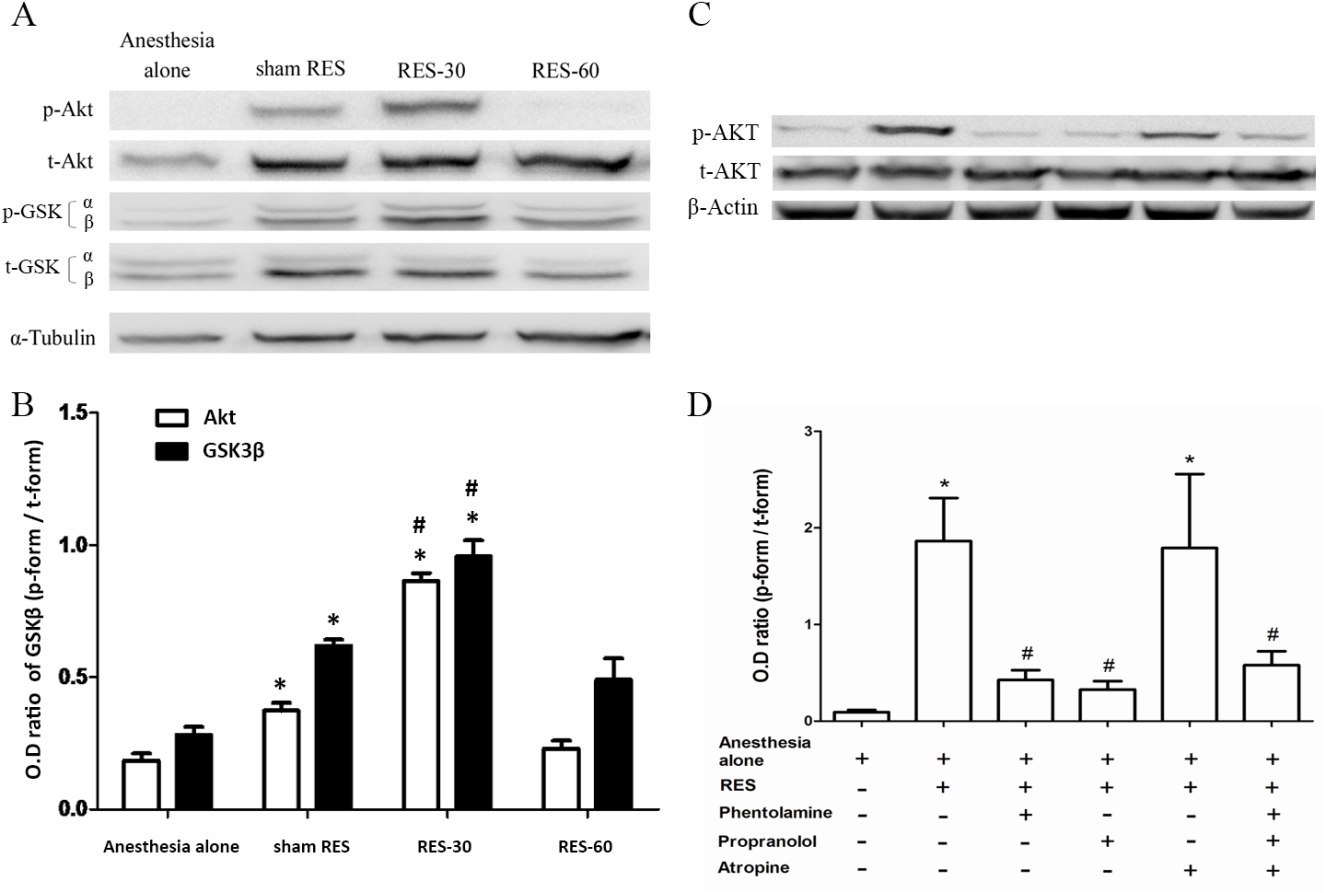
The role of α-adrenergic receptor, β-adrenergic receptor and muscarinic receptor signaling in remote electro-stimulation (RES)-induced Akt phosphorylation. Rat hearts were harvested at the designated time points of 0 min, 30min and 60 min after RES preconditioning and then analyzed by Western blotting (A) followed by quantification (B). For mechanistic analysis, rat hearts pretreated with phentolamine, an α-adrenergic receptor antagonist, with propanolol, a β-adrenergic receptor antagonist, and with atropine, a muscarinic receptor antagonist, were harvested at 30 min after RES stimulation and the proteins were analyzed with Western blotting (C), followed by quantification (D). Optic density (OD) ratio = phosphorylated form divided by the total form. Only the band of GSK3-β was quantified. *, *p*<0.05, *vs*. anesthesia alone; #, *p*<0.05, *vs*. sham RES; n = 4 in each group.

To determine the effect of RES on Akt and GSK3 expression in rats, hearts were harvested at the specific time points (0 min, 30 min and 60 min) after RES on the area of the bilateral median nerve and tissue samples from these hearts were then analyzed by Western blotting. The results showed that RES-induced activation of the protein kinase B/Akt resulted a peak in activity at 30 min after RES, followed by a decline at 60 min after stimulation (Fig 2A and 2B, n = 4); this activation was blocked by pretreatment with phentolamine (an α- adrenergic receptor antagonist) and by propanolol (a β-adrenergic receptor antagonist), but not by atropine (a muscarinic receptor antagonist). These findings suggest that both adrenergic receptors play a role in RES-induced Akt activation (Fig 2C and 2D, n = 4).

### The role of opioid receptors signaling in RES-induced myocardial GSK3 and PKCε expression

**Fig 3:**
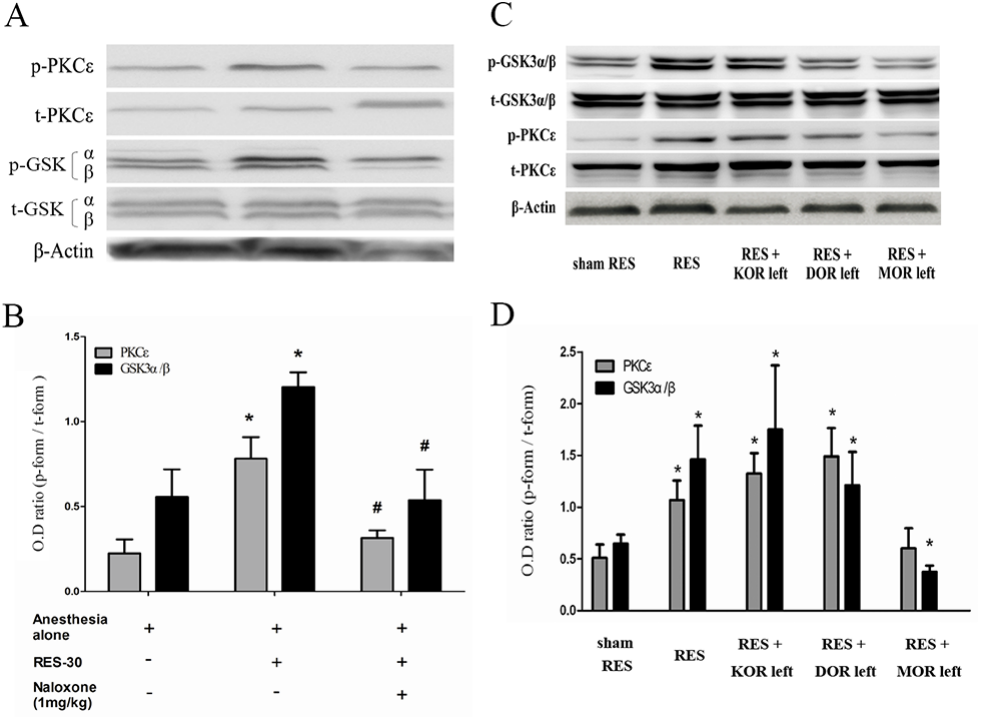
The role of opioid receptors signaling in remote electro-stimulation (RES)-induced myocardial GSK3 and PKCε expression. A non-selective opioid receptor antagonist, naloxone (1 mg/kg) (A, B, n = 4–5), and three specific opioid receptor subtypes antagonists targeting the kappa opioid receptors (KORs), delta opioid receptors (DOR)s and mu opioid receptor (MOR) antagonists (C, D, n = 4–5), were used to pretreat the animals for 15 min before RES preconditioning. Next, after RES treatment for 30 min, the heart proteins were analyzed by Western blotting. Optic density (OD) ratio = phosphorylated form divided by the total form. Only the band of GSK3-β was quantified. *, *p*<0.05, *vs*. sham RES; #, *p*<0.05, *vs*. RES-30 or RES. KOR, kappa opioid receptor; DOR, delta opioid receptor; MOR, mu opioid receptor; KOR left, KOR activity remained; DOR left, DOR activity remained; MOR left, MOR activity remained.

To investigate the role of opioid receptors signaling in RES-induced myocardial GSK3 and PKCε activation, a non-selective opioid receptor antagonist, naloxone (1 mg/kg), was used to pretreat the animals for 15 min before RES on bil. median nerves. The results showed that RES-induced GSK3 and PKCε phosphorylation was blocked by naloxone treatment (Fig 3A and 3B, n = 4–5), which suggests the opioid receptor signaling also plays a role in GSK3 and PKCε phosphorylation.

Next we pretreated the animals with three different antagonists that individually targeted various opioid receptor subtypes, namely, the kappa opioid receptor (KOR), the delta opioid receptor (DOR) and the mu opioid receptor (MOR) antagonists; treatment was carried out for 15 min before RES and the tissue samples were examined by Western blotting in order to identify GSK3 and PKCε activation. The results showed that the RES-induced GSK3 and PKCε phosphorylation was, firstly, significantly blocked by pretreatment with the KOR/DOR antagonists, which left MOR active. Secondly, EA-induced GSK3 and PKCε phosphorylation was, partial blocked by treatment with KOR/MOR antagonists, which left DOR active. Finally, RES-induced GSK3 and PKCε phosphorylation was not blocked by treatment with DOR/MOR antagonists, which left KOR active. These findings suggest that the roles of the various opioid receptor subtypes in RES-induced GSK3 and PKCε phosphorylation vary and have the importance KOR > DOR > MOR (n = 4–5 in each group).

### The effects of different opioid receptors subtype in preconditioned RES-induced myocardial protection against I/R injury

**Fig 4:**
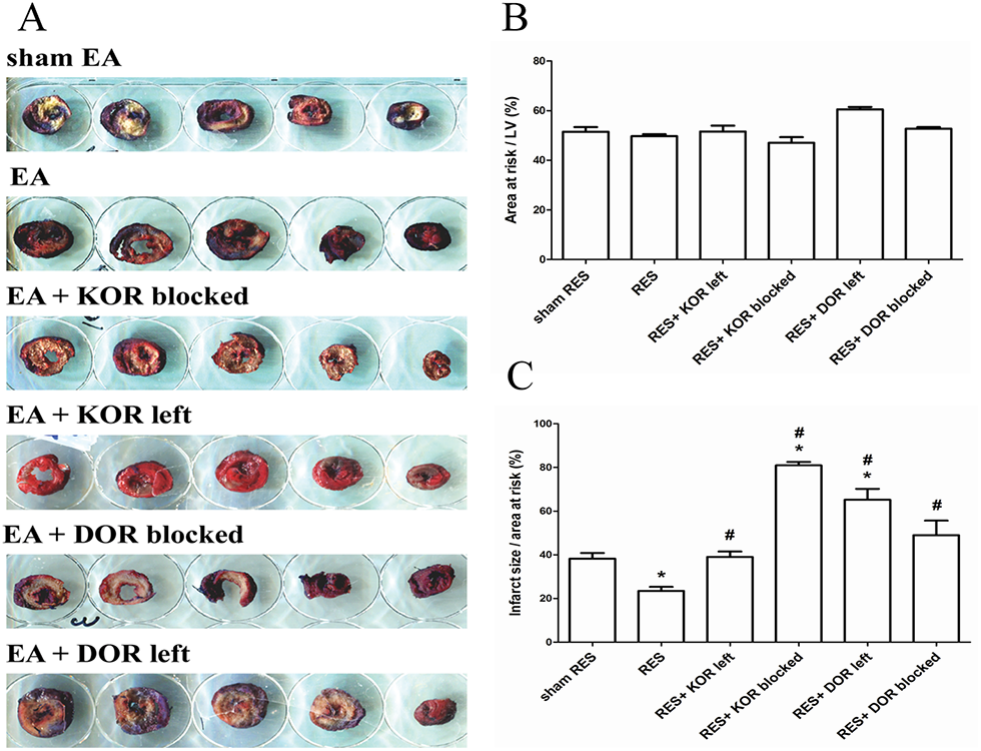
The role of different opioid receptors subtype affecting infarction size in preconditioned remote electro-stimulation (RES)-induced myocardial protection against ischemia/reperfusion (I/R) injury. In the I/R model, animals were randomly allocated into 6 groups as described in Methods. They were 1) sham RES group (n = 24); 2) RES preconditioning group (n = 20); 3) RES preconditioning pretreated with KOR blocker (n = 18); 4) RES preconditioning pretreated with DOR blocker (n = 10); 5) RES preconditioning pretreated with KOR/MOR antagonists, which left DOR active (n = 11); 6) RES preconditioning pretreated with DOR/MOR antagonists, which left KOR active (n = 9). All of these groups received subsequent I/R injury, followed by evaluation of infarct size, such as grossly (A), area at risk (B) and infarcted size (C). Data are presented as mean ± S.E.M and were analyzed using repeated one-way analysis of variance (ANOVA) followed by the Dunnet’s test. A *p* value less than 0.05 is considered statistically significant. *, *p*<0.05, *vs*. sham RES; #, *p*<0.05, *vs*. RES. KOR, kappa opioid receptor; DOR, delta opioid receptor; MOR, mu opioid receptor; KOR left, KOR activity remained; DOR left, DOR activity remained; MOR left, MOR activity remained.

In the I/R model, animals were randomly allocated into 6 groups. They were 1) sham RES group (n = 24); 2) RES preconditioning group (n = 20); 3) RES preconditioning pretreated with KOR blocker (n = 18); 4) RES preconditioning pretreated with DOR blocker (n = 10); 5) RES preconditioning pretreated with KOR/MOR antagonists, which left DOR active (n = 11); 6) RES preconditioning pretreated with DOR/MOR antagonists, which left KOR active (n = 9). All of these groups received subsequent I/R injury, followed by evaluation of infarct size, such as grossly (Fig 4A) and relative weight (Fig 4B and 4C). The results showed that there was no significant difference, in terms of area at risk, between groups (Fig 4B). Preconditioned RES (23±3%) significantly decreased the infacted size compared to sham RES group (41±5%), which was blocked by pretreatment of KOR and DOR antagonists, either alone or in combination (Fig 4C). The infarct size after KOR was blocked, KOR was left active, DOR was blocked and DOR was left active were 81±2%, 39±4%, 55±5%, and 65±9%, respectively.

### The effects of different opioid receptors subtype in hemodynamic changes of preconditioned RES-induced myocardial protection against I/R injury

**Fig 5:**
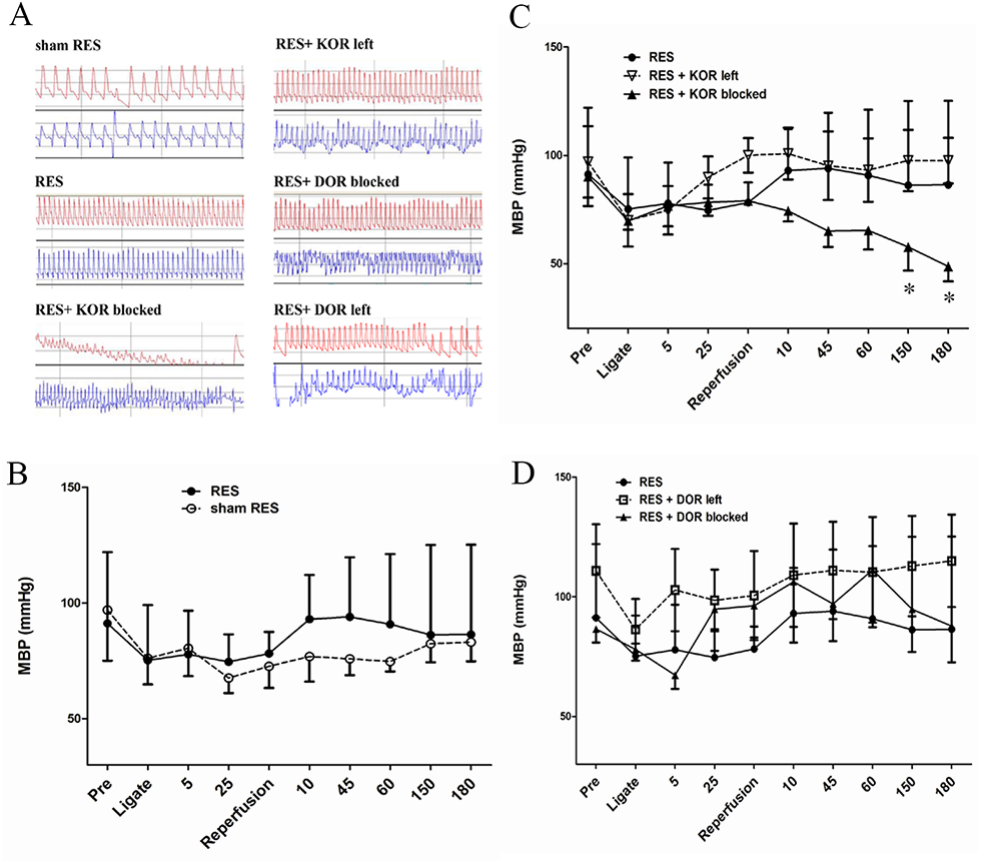
The role of different opioid receptors subtype in hemodynamic changes of preconditioned remote electro-stimulation (RES)-induced myocardial protection against ischemia/reperfusion (I/R) injury. Animals were randomly allocated into 6 groups as described in Methods. They were 1) sham RES group (n = 24); 2) RES preconditioning group (n = 20); 3) RES preconditioning pretreated with KOR blocker (n = 18); 4) RES preconditioning pretreated with DOR blocker (n = 10); 5) RES preconditioning pretreated with KOR/MOR antagonists, which left DOR active (n = 11); 6) RES preconditioning pretreated with DOR/MOR antagonists, which left KOR active (n = 9). During I/R injury period, the hemodynamic changes in these animals were continuously monitored (A). The results showed that preconditioned RES seemed to maintain a higher mean arterial pressure (MAP) than the sham RES group (B). It was of note that there was a significant decreased MAP in preconditioned RES + KOR blocked group (C), but not in preconditioned RES + DOR blocked group (D), compared to the preconditioned RES group. *, *p*<0.05, *vs*. sham RES. KOR, kappa opioid receptor; DOR, delta opioid receptor; MOR, mu opioid receptor; KOR left, KOR activity remained; DOR left, DOR activity remained; MOR left, MOR activity remained.

During I/R injury period, the hemodynamic changes in these animals were continuously monitored (Fig 5A). The results showed that preconditioned RES seemed to maintain a higher mean blood pressure (MBP) than the sham RES group (Fig 5B). It was of note that there was a significant decreased MBP in preconditioned RES + KOR blocked group (Fig 5C), but not in preconditioned RES + DOR blocked group (Fig 5D), compared to the preconditioned RES group.

### Preconditioned EA attenuated cardiomyocyte cell death and decrease mortality

**Fig 6:**
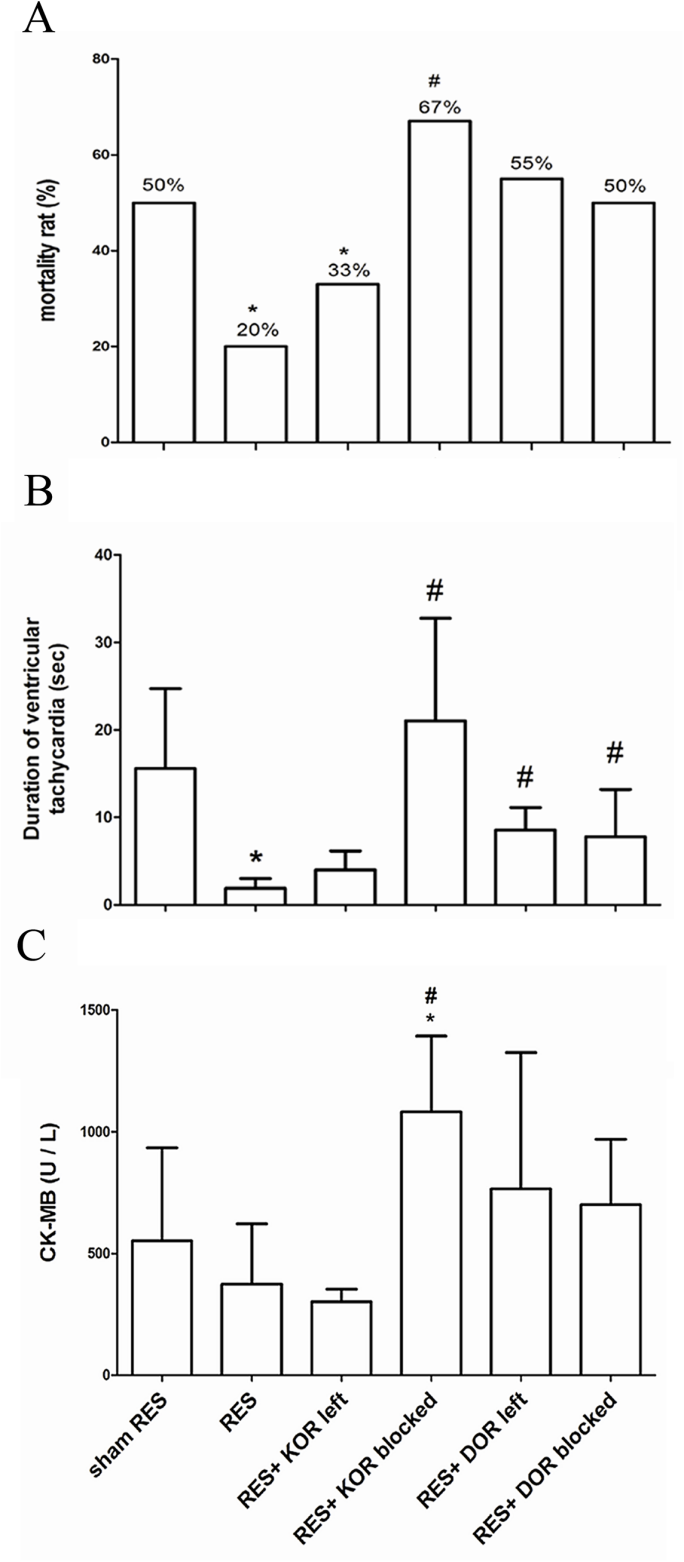
The role of different opioid receptors subtype affecting mortality in preconditioned remote electro-stimulation (RES)-induced myocardial protection against ischemia/reperfusion (I/R) injury. In the I/R model, animals were randomly allocated into 6 groups as described in Methods. They were 1) sham RES group (n = 24); 2) RES preconditioning group (n = 20); 3) RES preconditioning pretreated with KOR blocker (n = 18); 4) RES preconditioning pretreated with DOR blocker (n = 10); 5) RES preconditioning pretreated with KOR/MOR antagonists, which left DOR active (n = 11); 6) RES preconditioning pretreated with DOR/MOR antagonists, which left KOR active (n = 9). The mortality rate (A), duration of tachyarrhythmia (B), CK-MB (C) and troponin I (D) were evaluated during I/R injury. Data are presented as mean ± S.E.M and were analyzed using repeated one-way analysis of variance (ANOVA) followed by the Dunnet’s test. Mortality rate was analyzed using Fisher’s exact test. A *p* value less than 0.05 is considered statistically significant. *, *p*<0.05, *vs*. sham RES; #, *p*<0.05, *vs*. RES. KOR, kappa opioid receptor; DOR, delta opioid receptor; MOR, mu opioid receptor. KOR left, KOR activity remained; DOR left, DOR activity remained; MOR left, MOR activity remained.

All rats that had successful undergone ligation and relaxation of the coronary arteries were initially included in the study. Among these, five died due to anesthetic accidents. The mortality of the RES alone group (20%) was significant lower than that of the sham RES group (50%). In addition, opioid status as a result of pretreatment with various OR antagonists, either alone or in combination, was shown to affect the outcome of I/R injury. The mortality rate after KOR was blocked, KOR was left active, DOR was blocked and DOR was left active were 67%, 33%, 50% and 55%, respectively.(Fig 6A). Severe arrhythmia, including ventricular tachycardia (VT) and ventricular fibrillation (VF), was observed in the sham RES group, while the duration of the ventricular arrhythmia was significantly smaller and shorter, in preconditioned RES group. It should be noted that the duration of ventricular arrhythmia (Fig 6B) and serum CK-MB (Fig 6C), were significant increase in the group where KOR was blocked compared to the preconditioned RES group. These findings suggested that the high incidence of ventricular arrhythmia and low MAP might contribute to the high mortality found to affect the KOR blockaded group.

### Molecular evidence of KOR in rat heart

**Fig 7:**
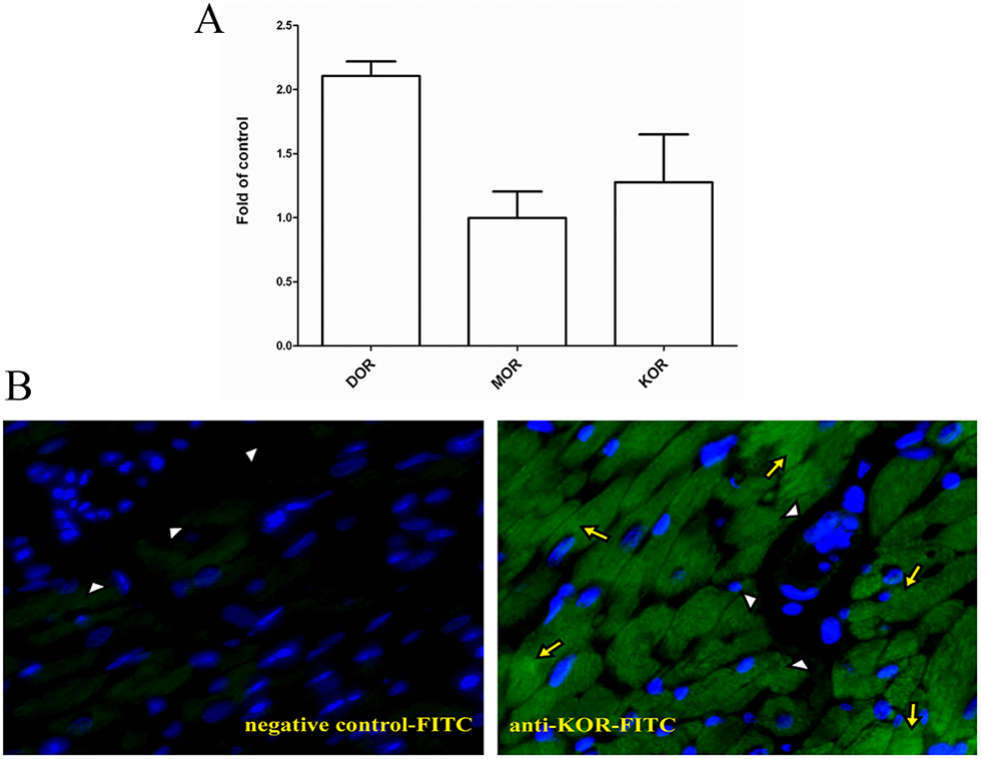
Molecular evidence of kappa opioid receptor (KOR) in rat heart. After rat hearts were harvested from the animals, total RNA was isolated and tissue specimen prepared for real-time PCR (A) and immunofluorecent staining (B) as described in Methods. The real-time PCR was analyzed using glyceraldehyde-3-phosphate dehydrogenase (G3PDH) as internal control and normalized using mu opioid receptor as relative baseline level. KOR, kappa opioid receptor; DOR, delta opioid receptor; MOR, mu opioid receptor. Yellow arrows indicate the localization of KOR in cardiomyotes while white arrow heads indicate the margin of a vessel. The results were repeated in three independent experiments, only represented figure was shown.

To validate the presence of KOR in rat heart, real-time PCR and immunostaining were performed. The results showed that the level of mRNA transcript of ORs in rat heart (Fig 7A), and Fig 7B demonstrated the localization of KOR in rat myocardiocytes, indicating KOR was *de novo* synthesized.

## Discussion

In this study, we demonstrated that preconditioned RES protects rat hearts against I/R injury via an activation of the myocardial opioid receptor signaling pathway. To our knowledge, we are the first to elucidate the relationship between GSK3, the opioid receptors and RES-induced myocardial protection.

There is consensus that activation of the kinase B (PKB) or Akt, which is known to be a pro-survival signaling, plays an important role in myocardial protection; this has been shown to occur via GSK3 signaling and acts to limit damage due to events such as ischemia-reperfusion (I/R) injury [35, 36]. When GSK3 is phosphorylated by protein kinase B (Akt), this leads to non-pathological cardiac hypertrophy; such hypertrophy results in an increase in the muscle mass of the heart together with a greater pumping ability [8, 37]. Although activation of cardiac phosphoinositide 3-kinase (PI3K) by growth factors such as insulin is known to stimulate the activity of the kinase Akt, which in turn phosphorylates and inhibits GSK3 activity, recent investigation have suggested that cytokine- stimulated phosphorylation of GSK3 is primarily dependent on PKC signaling rather than PKB signaling [38]. In addition, any inhibition of GSK-3β activity by Wnt or another pathway has been shown to result in stabilization of β-catenin and its translocation from the cytosol into the nucleus. This is where it partners with T-cell factor (TCF) and lymphoid enhancer factor family (LEF) members, allowing it to induce transactivation of a range of genes that contain TCF/LEF1 binding elements in their promoters [39]. Our results showed that preconditioned RES was able to activate the kinase Akt producing a peak at 30 min, followed by a decline at 60 min. These changes were found to be blocked by pretreated with phentolamine and propanolol, which suggests the adrenergic receptors have a role in RES-induced Akt activation. It is of note that adrenergic signaling and cholinergic singling induce opposite effect on Akt activation. We did not block neither adrenergic nor cholinergic receptors when we studied the role of opioid receptor signaling in RES-induced cardioprotection. Therefore, the Akt activation plays a lesser role in GSK3 phosphorylation in this setting.

There are three opioid receptor subtypes, μ, κ and δ, each of which has their own endogenous ligand, namely endomorphin, enkephalin, and dynorphin, respectively. It is well known that the mechanisms of acupuncture analgesia have been postulated to involve the mobilization of various central neuropeptides via distinct peripheral stimulation frequencies [40]. The opioid receptors can not only be observed in the brain and spinal cord, but are also present in the heart. Previous studies demonstrated that activation of the δ-opioid receptors was involved in ischemia preconditioning [19, 41] and in the protection of the heart against post-ischemic contractile dysfunction [22]. This possibly occurs via a protein kinase C (PKC)-dependent pathway [21]. Recent evidence revealed that κ-opioid receptors play an important role in cardioprotection via modulation of TLR4/NF-κB signaling and remote ischemic preconditioning [42, 43]. Our PCR results provided evidence of the presence of opioid receptor subtype mRNA transcripts, which agreed with a number of earlier reports on rats [43, 44] and on humans [45]. Although previous study proposed that the central κ opioid receptors are activated by peripheral stimulation with 100 Hz [13], our results demonstrated that the κ and δ opioid receptors were activated by RES stimuli at the frequency of 2/15 Hz, alternatively. Previously, Peart JN et al. demonstrated that KOR affords cardioprotection when activated prior to, but not after, reperfusion [46], which is inconsistent with our findings that RES protects rat hearts against I/R injury via prior activation of opioid receptors signaling pathway with the order of opioid receptor subtypes to be κ-receptors >δ-receptors >μ- receptors.

It has been found that activation of the opioid, adenosine, bradykinin, adrenergic and other G-protein coupled receptors is cardioprotective. Among the various opioid receptor subtypes, it has been found that activation of the κ- opioid receptors and/or the δ-opioid receptors was involved in direct myocardial protection [19]. Similarly, acupuncture is known to have a modulatory effect on the human cardiovascular system in areas such as antihypertension and the autonomic system [47, 48]. Accumulating evidence using animals has suggested that RES inhibition of cardiovascular sympathetic neurons, which has occurred via activation of visceral reflex stimulation, occurs through the activation of neurons in the arcuate nucleus of the hypothalamus, through the activation of the vlPAG in the midbrain and through the activation of the NRP in the medulla. These changes, in turn, inhibit the activity of premotor sympathetic neurons in the rVLM [26, 49]. Our previous studies have demonstrated that RES protects rats against I/R injury of the heart via a decrease in blood pressure and a reduced oxygen demand [29]. Taken together, the mechanisms by which acupuncture protects the heart against I/R injury seems to be via a multiplicity of targeted signaling systems, including PKB/Akt, PI3K, the κ- opioid receptor-PKC pathway and/or the δ-opioid receptor-PKC pathway. In conclusion, our results provide important information that should help to improve both the prevention and treatment of ischemia heart disease.

## Supporting Information

S1 Table The ARRIVE Guidelines Check List. (PDF)

## Abbreviations

RES: remote electro-stimulation
OR: opioid receptor
KOR: kappa opioid receptor
DOR: delta opioid receptor
MOR: mu opioid receptor
KOR: left, KOR activity remained
DOR: left, DOR activity remained
MOR: left, MOR activity remained
